# The Effect of Antibiotic Treatment of Early Childhood Shigellosis on Long-Term Prevalence of Attention Deficit/Hyperactivity Disorder

**DOI:** 10.3390/children8100880

**Published:** 2021-10-02

**Authors:** Yair Sadaka, Judah Freedman, Shai Ashkenazi, Shlomo Vinker, Avivit Golan-Cohen, Ilan Green, Ariel Israel, Alal Eran, Eugene Merzon

**Affiliations:** 1 The Joyce and Irving Goldman Medical School, Faculty of Health Sciences, Ben-Gurion University of the Negev, Beer-Sheva 8443944, Israel; yair.sadaka@gmail.com (Y.S.); judefreedman@gmail.com (J.F.); 2 Neuro-Developmental Research Center, Mental Health Institute, Beer Sheva 8461144, Israel; 3 The Adelson School of Medicine, Ariel University, Ariel 40700, Israel; shaias@ariel.ac.il; 4 Leumit Health Services, Tel-Aviv 6473817, Israel; svinker@leumit.co.il (S.V.); agolanchoen@leumit.co.il (A.G.-C.); aisrael@leumit.co.il (A.I.); 5 Department of Family Medicine, Sackler School of Medicine, Tel Aviv University, Tel Aviv 6997801, Israel; igreen@leumit.co.il; 6 Department of Life Sciences, Ben Gurion University, Beer Sheva 8443944, Israel; Alal_Eran@hms.harvard.edu; 7 Computational Health Informatics Program, Boston Children’s Hospital, Boston, MA 02115, USA

**Keywords:** *Shigella*, ADHD, antibiotics, neurodevelopment, microbiome

## Abstract

It has recently been shown that children with early shigellosis are at increased risk of attention deficit/hyperactivity disorder (ADHD). This study aimed to evaluate the association between antibiotic treatment of shigellosis with long-term ADHD rates. A retrospective cohort study was conducted that included all the Leumit Health Services (LHS) enrollees aged 5–18 years between 2000–2018 with a documented *Shigella*-positive gastroenteritis before the age of 3 years. Of the 5176 children who were positive for *Shigella* gastroenteritis before the age of 3 years, 972 (18.8%) were treated with antibiotics early (<5 days), 250 (4.8%) were treated late (≥5 days), and 3954 children (76.4%) were not prescribed antibiotics. Late antibiotic treatment was associated with significantly increased rates of ADHD (adjusted OR = 1.61; 95% CI, 1.1–2.3). Early treatment with antibiotics was not associated with increased ADHD rates (adjusted OR = 1.02; 95% CI, 0.8–1.3). In conclusion, late antibiotic treatment of early childhood shigellosis was associated with increased rates of ADHD.

## 1. Introduction

*Shigella* spp. cause acute gastroenteritis, sometimes severe, with high fever, bloody/mucous diarrhea, lower abdominal cramps, tenesmus, and severe toxicity [1]. Recent studies have shown that shigellosis is a significant worldwide cause of diarrhea and a leading cause of diarrheal death for children under the age of 5 years, especially in low- and middle-income countries [2].

In high-income countries, shigellosis is mostly a self-limiting gastrointestinal disease, with most patients recovering within 7–10 days. The infection, however, can lead to the loss of the colonic epithelial barrier function and severe inflammation [3]. Shigellosis is known to cause systemic complications, including short-term neurological manifestations, such as seizures and severe encephalopathy, related to brain inflammation and edemas that might be induced by the host’s response to toxin production [4,5,6].

Recently, a long-term neurodevelopmental complication has also been documented: a significantly higher rate of attention deficit/hyperactivity disorder (ADHD) was demonstrated in children who suffered from culture-proven *Shigella* gastroenteritis under the age of three years, the critical period of neurodevelopment, as compared to children without shigellosis [7]. It should be emphasized that ADHD is the most common childhood neurodevelopmental disorder that represents a common outcome pathway for a wide range of complex brain processes [8,9].

Antibiotic treatment of shigellosis considerably reduces the duration of fever, diarrhea, and pathogen shedding. It has also been shown that early antibiotic therapy reduces the excretion of Shiga toxins and probably also the toxin-mediated hemolytic–uremic syndrome (HUS) [10]. The guidelines of the World Health Organization (WHO) recommend antibiotic treatment for *Shigella* patients suffering from dysentery, although it seems that reliance on dysentery as the sole criterion for diagnosis may not be sensitive enough in detecting children at risk for disease complications. The Centers for Disease Control and Prevention (CDC) issued similar recommendations and suggest treating shigellosis if the patient is immunocompromised, has a severe illness, or is advised by health officials to take antibiotics for public health reasons [5].

It is unclear whether antibiotic treatment of shigellosis in children and its timing can reduce the risk of long-term neurodevelopmental disorders. To our knowledge, the relationship between the antibiotic treatment of shigellosis in early childhood and the risk of long-term neurodevelopmental disorders has not been reported. In this study, we aimed to explore the association between no antibiotic treatment, early antibiotic treatment, and late antibiotic treatment of *Shigella* infections and the risk of ADHD.

## 2. Materials and Methods

A cohort study was performed based on the study population which included all children aged 5–18 years who were registered with Leumit Health Services (LHS) between the years 2000 and 2018. LHS is one of the four major health maintenance organizations (HMOs) in Israel, which provided medical services to 724,129 enrollees during the study period. We used the LHS’s computerized database, which is updated with information including demographic characteristics, as well as medical information, such as physician visits, laboratory results, diagnoses, and prescriptions. The LHS’s database was described in detail in a previous study published by our group on the association between shigellosis and ADHD [7].

**Figure 1:**
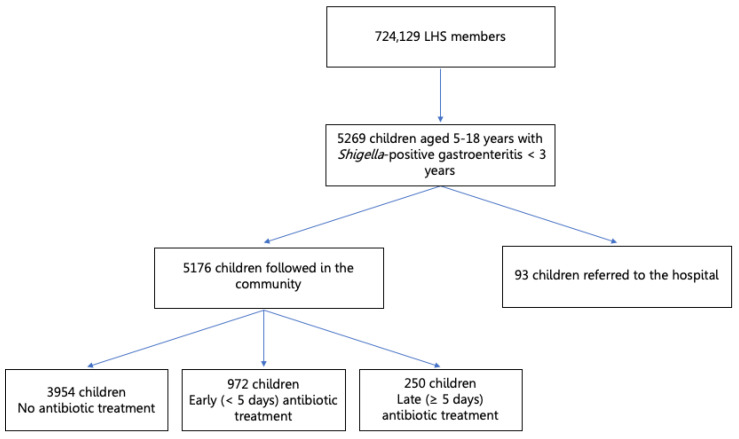
Study flowchart.

During the study period, there were 52,761 children aged 5–18 years who were LHS enrollees. Of these, 5269 children had at least one *Shigella*-positive stool culture during an episode of acute gastroenteritis before the age of 3 years; these children comprised the *Shigella* study group. In order to ensure that a more severe course of infection was not by itself the cause of the neurological outcomes, 93 cases of severe infection defined as children who were referred to the emergency department (ED) or hospitalized due to a *Shigella* infection were excluded from the analysis (Figure 1).

It should be clarified that not all children with acute diarrhea are referred for a stool culture. The decision to send a child for a stool culture is based on the physician’s clinical opinion and is often reserved for cases of moderate–severe gastroenteritis or dysentery, as is usually recommended in the Israeli and international guidelines. Stool specimens for culture were examined on the *Shigella/Salmonella* agar with selenite enrichment at 37 °C in the LHS’s central microbiologic laboratory according to the common protocols.

Dependent variable: Diagnosis of ADHD was identified with ICD-9 codes (314.00–314.9). To ensure the diagnosis of ADHD, only the children utilizing three prescriptions or more of anatomical therapeutic chemical (ATC) code N06B (psychostimulant agents and nootropics) used for ADHD treatment were included in the outcome.

Independent variable: To create the variable timing of antibiotic treatment, first, the crude odds ratio (OR) and the 95% confidence interval (95% CI) were calculated for the children who began antibiotic treatment on each day following the first visit with the disease and compared to those of the children who received no antibiotics while excluding the children who began antibiotic treatment on other days. Based on this analysis, the timing of antibiotic treatment was then split dichotomously from the time of the initial pediatrician visit into early (<5 days) and late (≥5 days) antibiotic treatment. We recorded and analyzed the specific antimicrobial agents that were prescribed.

Confounder variables: Four age groups were defined: early infancy (0–3 months), late infancy (4–12 months), second year (13–24 months), and third year (25–36 months). Socioeconomic status (SES) was defined according to the child’s home address using the Israeli Central Bureau of Statistics classification that includes 20 subgroups. Classifications 1–7 are considered to indicate low SES, 8–13—middle SES, and 14–20—high SES.

Ethnicity was defined according to the child’s home address as follows: secular Jewish, orthodox Jewish, and Israeli Arabs.

### Statistical Analysis

Statistical analysis was performed using IBM SPSS Statistics version 26. Assumptions were two-sided, with an α of 0.05. The initial analysis compared demographic characteristics between the subgroups of the study (*Shigella*-positive children treated early with antibiotics vs. *Shigella*-positive children treated late with antibiotics vs. *Shigella*-positive children not treated with antibiotics) using the Fisher’s exact χ2 test’s categorical variables (gender, age categories, ethnicity, and SES). Categorical data are shown in counts and percentages.

The primary outcome was ADHD amongst the children who were treated early or late with antibiotics for a *Shigella*-positive culture as compared to the children who had a *Shigella*-positive culture but were not treated with antibiotics. Univariable and multivariable logistic regression was used to estimate the crude and adjusted odds ratios (adjusted OR) and 95% CI for the independent association between antibiotic treatment for *Shigella* gastroenteritis and ADHD while controlling for potential confounders such as gender and socioeconomic status that have been recognized as potential risk factors for ADHD.

The study was approved by the Institutional Research Committee of LHS; as this was a data-based study, no clinical trial registration number was required. The study received IRB approval from the Shamir Medical Centre’s IRB (0005-18-LEU).

## 3. Results

### 3.1. Study Population

Of the 5176 children who were LHS members during the study period, had at least one positive stool culture for *Shigella* spp. before 3 years of age, and who were not hospitalized or referred to the ED due to a *Shigella* infection, 1222 were treated with antibiotics: 972 were treated early, within 5 days from the first visit with the disease, and 250 were treated late—5 days from the first visit or later. The remaining 3954 children were not prescribed antibiotics. Table 1 compares the demographic data of the study groups. Differences in age at the time of the *Shigella* infection, gender, ethnicity, and SES were all statistically insignificant between the study groups.

### 3.2. Univariable Analysis

**Figure 2:**
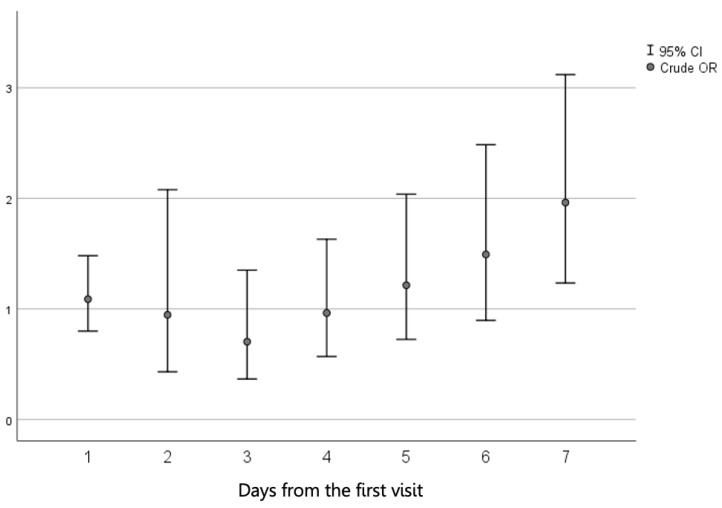
Crude odds ratio (OR) and 95% confidence interval (95% CI) of the association between the time (in days) between the first visit and the initiation of antibiotic treatment for shigellosis and ADHD rates as compared to no antibiotic treatment.

Initially, the cutoff for early versus late antibiotic treatment was defined. Using the univariable analysis, the crude ORs and 95% Cis were calculated for the children who began antibiotic treatment on each day following the first visit and compared to those of the children who received no antibiotics while excluding the children who began antibiotic treatment on other days. As demonstrated in Figure 2, beginning antibiotic treatment on the 5th day post-visit onwards was associated with a gradual increase in the crude OR. Therefore, the timing of antibiotic treatment was then split dichotomously: early antibiotic treatment was defined as treatment that started within five days of the initial visit and late antibiotic treatment was considered if treatment began five days after the initial visit or later.

Late antibiotic treatment at 5 days after the initial clinic visit or later was significantly associated with a higher rate of ADHD, with a crude OR of 1.72 (95% CI, 1.21–2.44, *p* = 0.002) as compared to the children who received no antibiotic treatment. Early antibiotic treatment (within the first 5 days from the clinic visit) was not associated with an increased risk of ADHD (*p =* 0.873).

Male gender was associated with increased risk of ADHD, with a crude OR of 2.01 (95% CI, 1.67–2.45, *p* < 0.001). As compared to the children of the lowest SES (reference group), children of the middle SES had a crude OR of 1.35 (95% CI, 1.10–1.63, *p* = 0.003), and children of the high SES had a crude OR of 1.55 (1.13–2.14, *p* = 0.006). No significant association was found between the age at the time of the *Shigella* infection and ADHD (Table 2). Regarding the type of antimicrobial agents used, most children were treated with azithromycin (an advanced macrolide) or third-generation cephalosporines, and a minority—with beta-lactam-like amoxycillin clavulanate. There was no significant difference in ADHD rates between the various groups of antibiotic agents prescribed—macrolides, cephalosporins, or beta-lactams (*p* = 0.276).

### 3.3. Multivariable Analysis

For the multivariable analysis, we incorporated all the variables that were significant in the univariable analysis (*p* < 0.1). Age at the time of positive *Shigella* culture, though insignificant, was used in the final model due to the clinical importance of this variable on the main outcome. Ethnicity was associated with a high degree of multicollinearity with the variable “socioeconomic status” and was therefore not included in the final model. Realizing that most children with ADHD were of Jewish ethnicity, we chose SES as a more valuable confounder to adjust for in the final model.

After controlling for gender, SES, and age at the time of positive culture, a significant independent association between late antibiotic treatment and ADHD was documented (adjusted OR = 1.61; 95% CI, 1.10–2.37; *p* = 0.015). Male gender (adjusted OR = 2.08; 95% CI, 1.70–2.37; *p* < 0.001), and both middle (adjusted OR = 1.40; 95% CI, 1.15–2.55; *p* = 0.001) and high SES (adjusted OR = 1.16–2.20, *p* = 0.004) were also associated with an increased risk of ADHD. Early treatment with antibiotics and younger age at the time of positive culture were not significantly associated with ADHD (Table 3).

## 4. Discussion

The association between *Shigella* gastroenteritis at a young age and long-term neurodevelopmental complications has been previously established. The rate of ADHD was 10.6% among the children with *Shigella*-positive gastroenteritis as compared to 8.6% among the children with *Shigella*-negative stool cultures [7]. The rate of ADHD among the children who had shigellosis below the age of three years was above the prevalence range of ADHD in Israeli children, which is between 7.4% and 9.5% [7]. In this study, we made an additional step and examined the association between antibiotic treatment for early childhood *Shigella* infection and the risk of future ADHD. The current results highlighted that the timing of antibiotic administration was crucial: the children who received antibiotics late (5 days from the initial visit with the infection or later) were at significantly increased risk of developing ADHD when compared to the children who did not receive antibiotic treatment. On the other hand, early antibiotic treatment was not associated with either an increased or decreased risk of ADHD as compared to no antibiotic treatment.

In our cohort of children with culture-confirmed *Shigella* gastroenteritis, 76.4% did not receive antibiotic treatment. These were probably children with a mild disease, for whom antibiotics are usually not recommended as their illness is typically self-limiting, especially in high-income countries [5,11]; they are probably at lower risk of complications, including long-term neurodevelopmental sequelae such as ADHD. The children who received antibiotics probably presented with a more severe symptomatic disease or a prolonged course as a reason for initiating antibiotic treatment and thus were at increased risk for short-term as well as long-term complications.

The effect of antibiotic treatment and its timing on the risk of ADHD can be mediated by several potential avenues. On the one hand, antibiotic treatment kills bacteria and therefore reduces toxin production, thus decreasing the duration of symptoms with associated inflammation, as documented with Shiga toxins in Bangladesh [10]; the reduced brain inflammation decreases the risk of ADHD. On the other hand, antibiotic administration has a significant and prolonged influence on the human microbiome, which by itself affects the rates of neurodevelopmental disorders, irrespective of the role of the *Shigella* infection [12,13]. These two avenues, together with the course of the *Shigella* infection, may have played a role in our patients.

Based on these pathogenic mechanisms, we can attempt to clarify the crucial distinct effects of antibiotic treatment according to the timing of its administration. Children who received early antibiotic treatment (within 5 days of the initial clinic visit) were not at higher risk for ADHD compared to those who did not receive antibiotics. However, as the former group probably had a more severe disease, it is plausible that early antibiotic treatment reduced the risk of ADHD by an early decrease of brain inflammation. Undoubtful confirmation of this explanation requires a randomized controlled study of antibiotics vs. a placebo for children with severe shigellosis, which is in fact not feasible because of the large sample size that would be needed in addition to being unethical as it has been well-documented that antibiotics enable a quick improvement of the symptoms related to childhood shigellosis.

On the other hand, the children who received late antibiotic treatment, after returning to their clinician for follow-up care, probably also had a severe or prolonged disease, which dictates the antibiotic administration. These children had a significantly increased risk of ADHD. Antibiotics had no significant protective effects in this scenario for several potential reasons. First, the acute neurological complications of shigellosis usually appear during the early course of the disease [11] so that brain inflammation with the late sequelae might also develop early. On the other hand, the late antibiotic administration had a significant effect on the human microbiome, which plays an important role in normal neurodevelopment [12,13]. Although the brain continues to develop well into adolescence, there is evidence that early childhood is the most important time of brain development. Within the first 2 years of life, the brain reaches 80–90% of the adult brain size [14]. The microbiome–gut–brain axis is a complex bidirectional axis modulated by the gut microbiota and affecting the immune, neural, endocrine, and metabolic pathways [15,16]. Both diarrheal illness and antibiotic treatment can cause a disequilibrium of the microbiome–gut–brain axis, and if these disturbances occur during the critical neurodevelopmental window, they can cause long-term neurological diseases [17]. Recent studies have shown the crucial role of the microbiome in normal neurodevelopment [18]. A disruption of the normal microbiome can affect various brain diseases, such as Alzheimer’s disease and Parkinson’s disease, by increasing the neuroinflammatory state [19,20]. There is also evidence associating the gut microbiome with neuropsychiatric disorders, such as depression, anxiety, and autistic spectrum disorders (ASD) [21,22].

Neurotoxic effects have been seen with several groups of antibiotics, including cephalosporins, penicillins, and macrolides. These effects include seizures, encephalopathy, and coma. Possible causes of neurotoxicity include blood–brain barrier penetration caused by prior CNS disease and altered drug pharmacokinetics caused by disturbed renal function [23,24]. Antibiotics have been shown to alter the normal microbiome of children by reducing its diversity and richness, increasing the presence of more virulent bacteria, and enhancing bacterial expression of antimicrobial genes [25]. Associations have been found between early treatment with antibiotics (within the first year of life) and neurocognitive outcomes, including a higher risk of parent- and teacher-reported rates of ADHD symptoms at the age of 11 years [26]. It is reasonable to believe that a longer, more severe *Shigella* infection combined with antibiotic treatment could have a synergistic effect on the level of dysbiosis, thus leading to harsher neurological outcomes, as indeed documented in our study.

ADHD is the most prevalent chronic neuropsychiatric disorder in children, with worldwide prevalence ranging between 5% and 10% [27]. Overall, 10.5% of our study population had a diagnosis of ADHD. This is consistent with the previous study which evaluated the entire pediatric LHS-insured population [7], although it is slightly higher than in the previous studies from Israel which showed a prevalence of 7.4% for Arab Israeli children and 9.5% for Jewish Israeli children [27].

Our study has a few limitations. It is a retrospective cohort study. However, despite this fact, by using patients’ data from the LHS’s database, we were able to create a large cohort which increased the study strength. By excluding children who were referred to the ED or hospitalized in addition to studying children who were insured by LHS throughout the study period, it is very unlikely that the children could have tested for stool culture elsewhere. In addition, our analysis lacked potential confounding variables, such as family history of ADHD, maternal smoking, and birth weight that might all have affected ADHD morbidity rates; these variables are probably distributed similarly among our study subgroups. Another limitation is our inability to provide data on the presentation and subtypes of ADHD and their relation to previous shigellosis due to the retrospective design of the study. Finally, we did not have information regarding the clinical presentation of shigellosis, although this information was presumed according to the therapeutic approach. Further studies are recommended in additional locations to assess the effect of severe illness and antibiotic treatment on the microbiome of young children suffering from a *Shigella* infection and to further analyze the association and causality of antibiotic treatment of the *Shigella* infection and ADHD.

## 5. Conclusions

Post-*Shigella* infection children are at increased risk for developing ADHD later in life. Our study shows that late antibiotic treatment of *Shigella* gastroenteritis in children younger than 3 years is associated with a significantly increased risk of ADHD as compared to no antibiotic treatment, independent of gender, SES, and age at the time of positive *Shigella* culture. This study supports the current guidelines for antibiotic treatment for severe *Shigella* infection and highlights the need for relatively early treatment in these cases. This study also represents an additional step towards understanding the complex relationships between the *Shigella* infection and probably other infections and late neurodevelopmental morbidity in these children, which might hopefully lead to efficacious preventive measures. Although more research is necessary, our study shows that early antibiotic treatment for *Shigella* gastroenteritis could probably reduce the risk of later ADHD, thereby improving the lives of millions of children worldwide, mainly in low-income countries.

## Figures and Tables

**Table 1:** Comparison of demographic variables between the study groups: no antibiotic treatment, early antibiotic treatment, and late antibiotic treatment.

Demographic Variables	Antibiotic Treatment for *Shigella* Gastroenteritis			*p*
	No Antibiotic Treatment Number (%) (*n* = 3954)	Early Antibiotic Treatment Number (%) (*n* = 972)	Late Antibiotic Treatment Number (%) (*n* = 250)	
Gender				
Male	2191 (55.4)	521 (53.6)	136 (54.4)	0.584
Female	1763 (44.6)	451 (46.4)	114 (45.6)	
Ethnicity				
Secular Jewish	2347 (59.4)	548 (56.4)	142 (56.8)	0.353
Orthodox Jewish	1269 (32.1)	344 (35.4)	84 (33.6)	
Israeli Arab	338 (8.5)	80 (8.2)	24 (9.6)	
SES				
High	323 (9.0)	79 (9.0)	21 (9.5)	0.084
Middle	1653 (46.1)	370 (42.0)	88 (39.6)	
Low	1607 (44.9)	432 (49.0)	113 (50.9)	
Age at positive stool culture examination				
0–3 months	659 (16.7)	148 (15.2)	41 (16.4)	0.626
4–12 months	1562 (39.5)	380 (39.1)	90 (36.0)	
13–24 months	1039 (26.3)	263 (27.1)	65 (26.0)	
25–36 months	694 (17.6)	181 (18.6)	54 (21.6)	

**Table 2:** Univariable analysis and crude odds ratio (OR) associating antibiotic treatment with *Shigella* gastroenteritis and ADHD (*n* = 5176).

Patient Variable		ADHD		*p*	Crude OR (95% CI) *p*
		No Number (%) (*n* = 4630)	Yes Number (%) (*n* = 546)		
Antibiotic treatment for *Shigella* infection	Treatment within 5 days of the clinic visit	871 (18.8)	101 (18.5)	0.008	1.01 (0.80–1.28) 0.873
	Treatment ≥ 5 days of the clinic visit	209 (4.5)	41 (7.5)		1.72 (1.21–2.44) 0.002
	No antibiotic treatment	3550 (76.7)	404 (74.0)		Reference group
Gender	Male	2467 (53.3)	381 (69.8)	<0.001	2.01 (1.67–2.45) <0.001
	Female	2163 (46.7)	165 (30.2)		
SES	High	367 (8.8)	56 (11.3)	0.002	1.55 (1.13–2.14) 0.006
	Middle	1864 (44.5)	247 (49.9)		1.35 (1.10–1.65) 0.003
	Low	1960 (46.8)	192 (38.8)		Reference group
Age at positive culture (months)	0–3 months	762 (16.5)	86 (15.8)	0.383	0.895 (0.662–1.21) 0.473
	4–12 months	1833 (39.6)	199 (36.4)		0.861 (0.67–1.10) 0.243
	13–24 months	1210 (26.1)	157 (28.8)		1.029 (0.79–1.33) 0.830
	25–36 months	825 (17.8)	104 (19)		Reference group

**Table 3:** Multivariable analysis with adjusted odds ratio (OR) and 95% confidence interval (95% CI) for the independent association between antibiotic treatment for *Shigella* gastroenteritis and ADHD (*n* = 4686).

Variable	Adj. OR	95% CI	*p*
Antibiotic treatment (<5 days vs. no treatment)	1.02	0.80–1.31	0.817
Antibiotic treatment (≥5 days vs. no treatment)	1.61	1.10–2.37	0.015
Gender (male vs. female)	2.08	1.70–2.55	< 0.001
SES (middle vs. low)	1.40	1.15–1.72	0.001
SES (high vs. low)	1.60	1.16–2.20	0.004
Age at positive *Shigella* culture (0–3 months vs. 25–36 months)	0.829	0.59–1.14	0.258
Age at positive *Shigella* culture (4–12 months vs. 25–36 months)	0.879	0.67–1.15	0.348
Age at positive *Shigella* culture (13–24 months vs. 25–36 months)	1.02	0.77–1.35	0.869

## Data Availability

We have full control of all the primary data and agree to allow the journal to review the data if requested.
